# Machine learning model predicts clotting risk during CRRT in ESKD patients: a SHAP-interpretable approach

**DOI:** 10.1080/0886022X.2025.2562448

**Published:** 2025-10-09

**Authors:** Shuang Qiu, Shibo Mu, Yongyuan Tao, Ning Zhang, Jiuxu Bai, Ning Cao

**Affiliations:** ^a^ Department of Blood Purification, General Hospital of Northern Theatre Command, Shenyang, China; ^b^ School of Mechanical Engineering, Shenyang University of Technology, Shenyang, China

**Keywords:** ESKD, continuous renal replacement therapy, prediction model, extracorporeal circuit clotting, machine learning

## Abstract

Ensuring fluent extracorporeal circulation and preventing circuit clotting are important for end-stage kidney disease (ESKD) patients undergoing continuous renal replacement therapy (CRRT). This study aimed to develop a predictive model using machine learning (ML) algorithms to evaluate clotting risk after initiating CRRT, enhancing treatment safety and effectiveness. This study involved 636 ESKD patients who underwent CRRT. Feature selection was conducted *via* the least absolute shrinkage and selection operator (LASSO) algorithm. ML algorithms, including support vector machine (SVM), extreme gradient boosting (XGBoost), random forest (RF), gradient boosting machine (GBM), decision tree, and logistic regression (LR), were applied to construct models through tenfold cross-validation. Model performance was assessed *via* the area under the receiver operating characteristic curve (AUC) and additional metrics. The Shapley additive explanation (SHAP) values quantify each feature’s contribution. This study included 199 patients with blood clots during extracorporeal circulation, corresponding to an incidence rate of 31.3%. The AUC values were 0.864 (SVM), 0.815 (XGBoost), 0.806 (GBM), 0.778 (RF), 0.732 (Decision Tree), and 0.717 (LR). The SVM exhibited the best performance. The initial dose of low-molecular-weight heparin (LMWH) was identified as the most significant factor influencing coagulation. ML serves as a reliable tool for predicting the risk of extracorporeal circuit clotting in ESKD patients undergoing CRRT. The SHAP method elucidates key risk factors, providing a basis for early clinical intervention.

## VISUAL ABSTRACT

**Figure:**
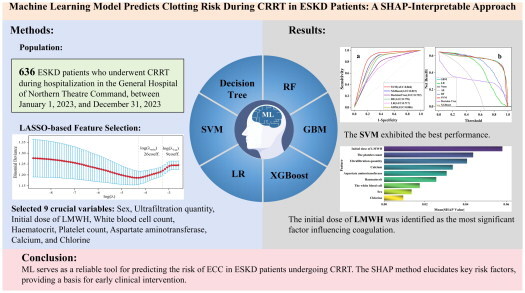

## Introduction

Chronic kidney disease (CKD) has become a global public health issue, with epidemiological data showing a median global prevalence rate of 9.5% [1]. As a terminal kidney disease, end-stage kidney disease (ESKD) has an incidence rate that increases from year to year, with the number of patients in China alone nearing 1.07 million, equivalent to approximately 600 individuals per million population relying on dialysis to sustain life, and it represents a significant threat to human health [2,3]. Among these patients, hemodialysis (HD) serves as the primary treatment method, benefiting up to 916,000 patients [4]. Its methods can be further classified into intermittent hemodialysis and continuous renal replacement therapy (CRRT) based on their distinct characteristics and indications [5–7]. Currently, research on CRRT primarily focuses on patients with acute kidney injury (AKI) in intensive care units. Epidemiological data indicates that approximately 50%–60% of intensive care units patients develop AKI [8], among which 10%–15% require CRRT treatment [5]. Notably, CRRT is also applicable for ESKD patients experiencing haemodynamic instability, multiple-organ dysfunction, hyperkalaemia, hypernatremia, severe metabolic acidosis, as well as those with cerebral edema or hypercatabolic states [9]. Although epidemiological data on CRRT treatment for ESKD patients is currently lacking, the number of critically ill ESKD patients is increasing annually, due to prolonged survival of dialysis patients, an increase in complications, and a rising incidence of CKD. Therefore, conducting relevant research on CRRT treatment for the ESKD population holds significant clinical importance.

A common problem encountered during CRRT is extracorporeal circuit clotting (ECC), which can disrupt treatment sessions, undermine treatment plans, reduce the efficiency of dialysis, cause blood loss, and increase the risk of thrombosis. These complications may lead to more vascular access issues and a higher risk of iatrogenic infections [10]. Additionally, such problems further increase the economic burden on patients and the workload for medical staff [11]. Consequently, it is of paramount importance to anticipate and intervene in potential coagulation issues within the extracorporeal circulation system at an early stage. The previously developed models for predicting ECC during CRRT, including those published by Fu et al. [12] using Cox proportional hazard-based regression analysis and Zhang et al. [13] employing LR analysis, have focused primarily on coagulation events in patients with AKI and those residing in intensive care units. To systematically compare the similarities and differences across various studies, we have summarized the key features of these models in Supplementary Table S1. However, there are significant differences in CRRT treatment strategies between AKI and ESKD patients, leading to distinct ECC risk factors. AKI patients are typically critically ill and require longer single-session dialysis, with regional citrate anticoagulation recommended by guidelines [14]. They also tend to have lower dialysis blood flow rates and larger replacement fluid volumes [8]. In contrast, ESKD patients require long-term dialysis, with shorter single-session durations, higher blood flow rates, and lower replacement fluid volumes [5]. Currently, there are no specific anticoagulation strategies recommended for ESKD patients. However, there is substantial evidence supporting the use of low-molecular-weight heparin (LMWH) in CRRT for ESKD patients [5,15]. Therefore, this study employed LMWH or no anticoagulation as the anticoagulation strategy, which aligns with current local clinical practice and helps minimize treatment variability. As anticoagulation strategies, dialysis blood flow, replacement fluid volumes, and CRRT treatment approaches all affect ECC, these factors need to be carefully considered. Nevertheless, there is currently no specific extracorporeal circulation coagulation prediction model tailored for ESKD patients. Therefore, the development of a dedicated extracorporeal circuit coagulation risk prediction model specifically for ESKD patients undergoing CRRT is urgently needed.

In recent years, the rapid advancement of machine learning (ML) technologies has led to remarkable performance in several clinical areas, especially in risk prediction, where they have shown immense potential and value [16–18]. In the field of critical care nephrology, ML is increasingly being applied to predict AKI, the need for dialysis, and associated complications [19]. Significant progress has been made in development of risk prediction models for ESKD patients and patients with AKI, and these approaches have demonstrated superior performance to traditional LR and Cox regression analyses [20–22]. Therefore, we aimed to investigate the applicability of ML to predict the coagulation status of extracorporeal circulation devices in ESKD patients undergoing CRRT while providing a comprehensive analysis of the associated risk factors.

## Methods

### Data source and study population

**Figure 1.**
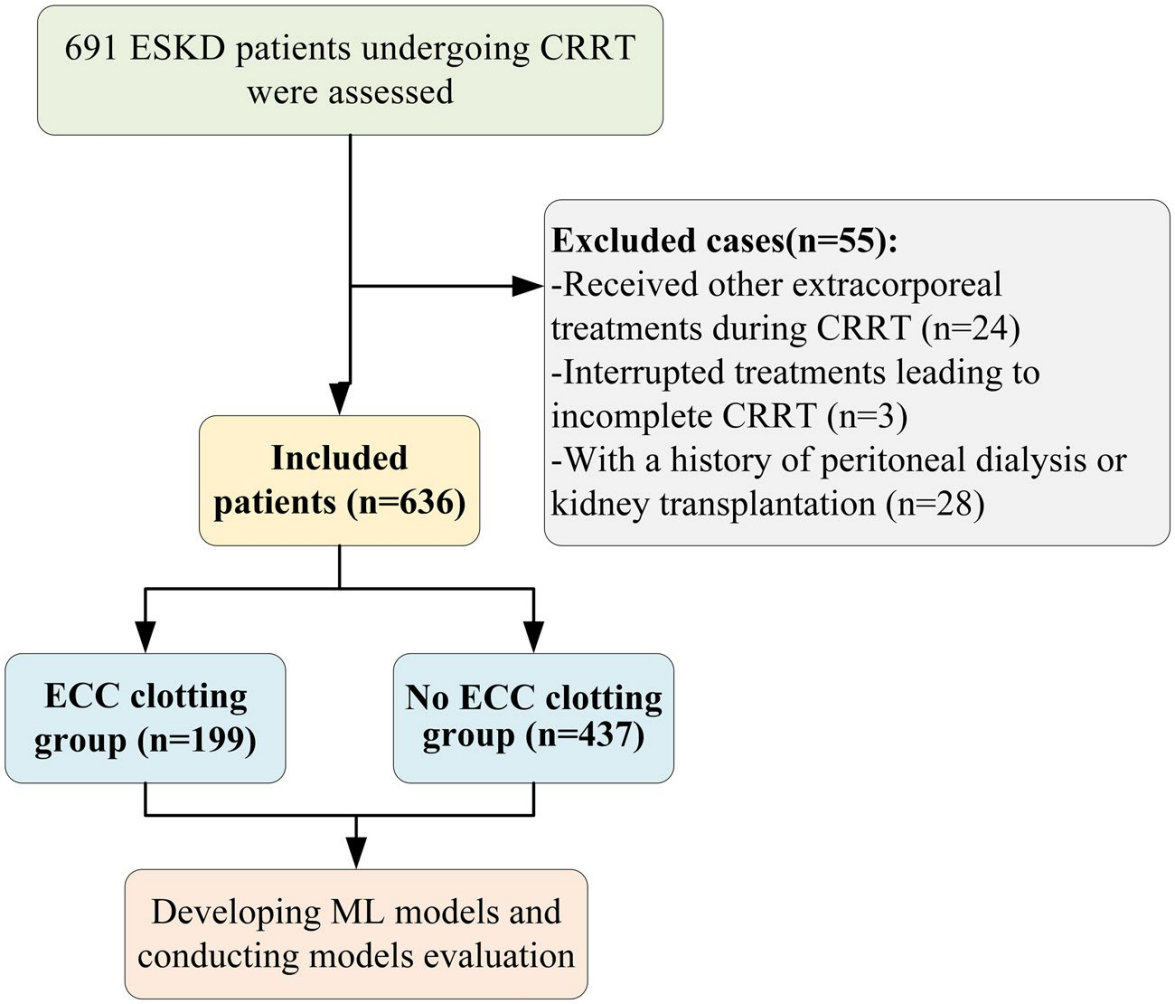
Flow chart of the study patients. Abbreviations: CRRT: continuous renal replacement therapy; ECC: extracorporeal circuit clotting; ML: machine learning.

This study retrospectively analyzed the medical records of 691 adult (≥18 years old) ESKD patients who underwent CRRT during hospitalization in the Department of Blood Purification, General Hospital of Northern Theater Command, between January 1, 2023, and December 31, 2023. ESKD is defined as an estimated glomerular filtration rate <15 mL/min/1.73 m^2^, which is calculated *via* the CKD-EPI formula, consistent with the KDIGO clinical practice guideline for the evaluation and management of CKD [5]. These patients had complete routine blood, coagulation, and biochemical test data available at the beginning of the CRRT process. This study also included patients who were unable to continue anticoagulation therapy due to bleeding events occurring during the study period. Patients who received other extracorporeal treatments (i.e. plasma exchange, haemoperfusion, or extracorporeal membrane oxygenation) during CRRT, those with interrupted treatments (including for death or other reasons) leading to incomplete CRRT, and those with a history of peritoneal dialysis or kidney transplantation were excluded. Ultimately, a total of 636 patients were included in the final analysis. Given that some patients underwent multiple CRRT sessions during hospitalization, to ensure the accuracy of the data and avoid duplicates, only the data from the first CRRT session of each patient were included in this study. ECC was defined as follows: (1) a sustained transmembrane pressure level above 300 mmHg (after excluding non-circuit clotting issues such as improper puncture, inadequate dialysis access, dialysis fluid flow problems, ultrafiltration volume, and machine malfunctions that may lead to elevated transmembrane pressure) and (2) the presence of grossly visible blood clots within the extracorporeal circuit [23], as shown in Figure 1. During CRRT, all participants were treated with the same continuous blood purification equipment and a standardized replacement fluid, ensuring the consistency and reliability of the treatment. This study protocol complied with the principles outlined in the Declaration of Helsinki, as revised in 2013, and was approved by the Ethics Committee of the General Hospital of Northern Theater Command (ethics approval number: Y-2024-231). This retrospective cohort study was designed and reported in accordance with the Strengthening the Reporting of Observational Studies in Epidemiology guidelines, ensuring transparency and methodological rigor in study design, data collection, analysis, and outcome reporting. The requirement to obtain written informed consent from each patient was waived because this was an observational retrospective study.

### Study variables

We collected general patient demographics, as well as CRRT treatment prescriptions and related data, and pre-CRRT laboratory data to establish and evaluate the performance of different models. The variables included demographic information (sex, age, and weight). The CRRT treatment prescriptions and related data included the type of blood dialysis filter (Gambro ST100, ST150, and M100), vascular access for dialysis included arteriovenous fistulas (cannulated using a 16 G needle, approximately equivalent to 1.65 mm in diameter), standard double-lumen internal jugular vein dialysis catheters (11.5 Fr, 13 cm), standard double-lumen femoral vein dialysis catheters (11.5 Fr, 20 cm), right internal jugular vein chronic catheter (tunneled, 14.5 Fr, 19 cm), left internal jugular vein chronic catheter (tunneled, 14.5 Fr, 23 cm), femoral vein chronic catheter (tunneled, 14.5 Fr, 33 cm), heart rate, average systolic blood pressure, average diastolic blood pressure, treatment model CVVH (continuous veno-venous haemofiltration), blood flow rate (150–250 mL/min) [24], replacement fluid (Yishuke brand, Shijiazhuang No. 4 Pharmaceutica, containing glucose, sodium chloride, magnesium chloride, and calcium chloride), ultrafiltration quantity, initial dose of LMWH (60–80 IU/kg) [5], hourly booster dose of LMWH (10–20 IU/kg/h) [5], or an additional dose of 20–25 IU/kg administered at the Xth hour. Additionally, all patients underwent a comprehensive baseline laboratory evaluation prior to initiation of CRRT. The reliability of these data sources was assured, with the CRRT treatment data originating from meticulously maintained paper records, which were cross-checked by a specialized medical team to ensure their accuracy. LMWH or no anticoagulation was used as the anticoagulation strategy. Additionally, laboratory data and general demographic information were sourced from the reputable laboratory center and inpatient management system of our institution, further enhancing the reliability and completeness of the data. To minimize data entry errors to the greatest extent possible, this study employed a rigorous dual-entry verification system.

### Statistical analysis

When performing the statistical analyses *via* R software (version 4.3.3; The Comprehensive R Archive Network; http://cran.r-project.org), we described the central tendencies and variability of the quantitative variables as the means ± standard deviations. For data that did not conform to a normal distribution, we chose the Wilcoxon rank-sum test to compare different groups. The categorical variables are presented as counts and percentages, and comparisons between these variables were performed *via* either the chi-square test or Fisher’s exact test, depending on factors such as the sample size and expected frequency.

## Data preprocessing

On the basis of the results of Little’s test for missing data completely at random, the data were found to be missing at random. To mitigate the bias caused by missing data, we used the multiple imputation [25] method to fill in gaps. This approach is based on chained equations, in which a predictive model is constructed for each missing variable. Through Monte Carlo simulations, multiple complete datasets were generated for statistical analysis purposes. The final results were then combined to produce more accurate statistical inferences. To evaluate the impact of data imputation on subsequent analytical reliability, we performed near-zero variance (sparsity) detection and multicollinearity assessment on the predictor variables.

Following missing value imputation, the synthetic minority oversampling technique (SMOTE) [26,27] was employed on the training set to counteract class imbalance in the classifier. SMOTE, which is an improvement over random oversampling algorithms, generates new samples by interpolating between minority class instances to increase their quantity. This process was carried out on the basis of the k-nearest neighbors of the minority class samples, which effectively balanced the dataset and improved the generalizability of the tested classification models. In this study, the optimal number of neighbors (*k* = 5) was determined through a grid search combined with cross-validation, using the area under the receiver operating characteristic curve (AUC) value as the evaluation metric. We subsequently employed the Kolmogorov–Smirnov test to evaluate the distribution characteristics of the post-SMOTE data.

## Feature selection

We employed the least absolute shrinkage and selection operator (LASSO) algorithm [28,29] to identify significant features, with the entire feature selection process strictly confined to the training set to prevent data leakage. By incorporating an L1 regularization term into the loss function, the LASSO algorithm both minimizes the prediction error and promotes sparsity in the model parameters. This strategy effectively compresses the coefficients of most features to zero, retaining nonzero coefficients for only a few crucial features, thereby enabling natural feature selection. The features corresponding to these nonzero coefficients had significant impacts on the target variable. LASSO not only simplified the models and improved their prediction accuracy but also effectively prevented overfitting by reducing the complexity of the models.

### Model development

After completing the feature selection step, we used five ML algorithms—support vector machine (SVM), extreme gradient boosting (XGBoost), random forest (RF), gradient boosting machine (GBM), decision tree, and traditional logistic regression (LR)—to construct our models. The adoption of these models was justified by their established effectiveness in classification tasks and proven ability to identify complex data patterns and relationships. To ensure the robustness and reliability of the model performance results, we adopted a 10-fold cross-validation strategy [30]. We divided the original dataset evenly into 10 balanced subsets and cycled through ten iterations. In each iteration, one subset served as an independent test set, whereas the remaining nine subsets were combined to serve as the training set. This approach ensured that every data subset had the opportunity to be used as a test set, thereby fully utilizing all the information contained in the full dataset and enhancing the generalizability of the models.

In our study, we used a radial basis function as the kernel for constructing the SVM model, where the optimal values of the regularization parameter (cost) and gamma were meticulously chosen through grid searches coupled with a 10-fold cross-validation strategy. To optimize the hyperparameters of XGBoost, we leveraged a 10-fold cross-validation approach to identify the optimal configurations for the number of iterations, maximum tree depth (max depth), learning rate (eta), subsample ratio (subsample), and feature sampling ratio (colsample_bytree). Similarly, for the RF model, we used 10-fold cross-validation to design its hyperparameters, setting key parameters such as ntree (the number of trees to grow), mtry (the number of randomly selected features at each split), and nodesize (the minimum number of samples required to split an internal node). In the case of the GBM, we also employed a 10-fold cross-validation strategy to determine its hyperparameters, including n.trees, interaction.depth (the maximum tree depth), and shrinkage (the learning rate). For the decision tree model, we leveraged the rpart.control function to set the model hyperparameters, incorporating the complexity (cp), maximum depth (maxdepth), minimum split size (minsplit), and number of cross-validation folds (xval). These hyperparameters were configured within the rpart.control function and passed to the rpart function *via* the control argument, ensuring that a high-quality decision tree model was trained. Finally, we constructed the LR model *via* the glm function, adopting the binomial (link = “logit”) function family to accommodate this binary classification problem. We tuned the hyperparameters through glm.control, specified the maximum number of iterations and convergence threshold, and employed a 10-fold cross-validation strategy to identify the optimal hyperparameter configuration.

### Model evaluation

**Figure 2.**
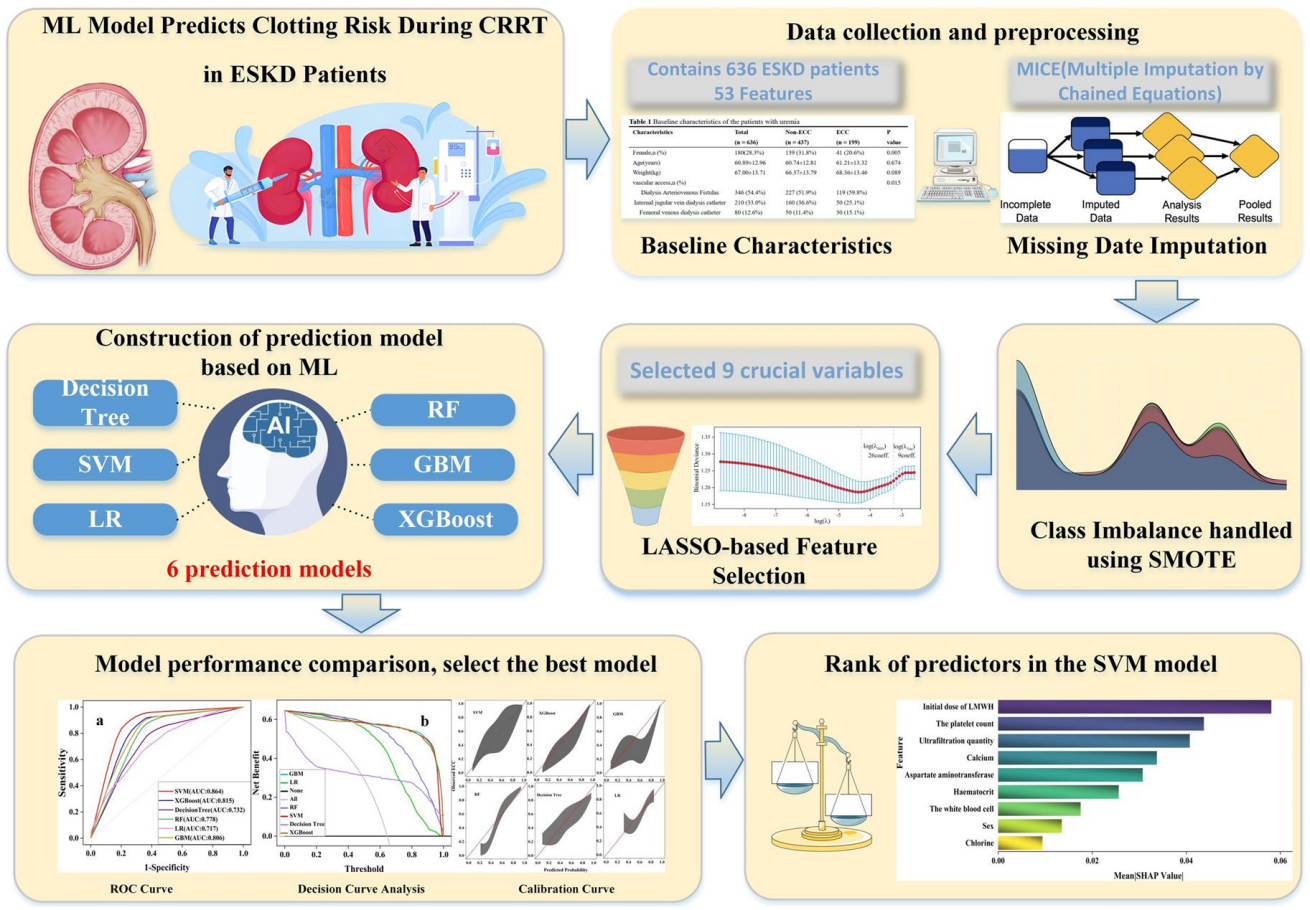
Study framework and feature engineering scheme. Abbreviations: ML: machine learning; CRRT: continuous renal replacement therapy; LASSO: least absolute shrinkage and selection operator; SMOTE: synthetic minority oversampling technique; SVM: support vector machine; XGBoost: extreme gradient boosting; RF: random Forest; GBM: gradient boosting machine; LR: logistic regression; DCA: decision curve analysis; ROC: receiver operating characteristic.

We calculated the AUC, sensitivity, Brier’s score, specificity, positive predictive value (PPV), negative predictive value (NPV), and F1 score to comprehensively evaluate the performance of each model [31]. Model interpretability was graded as High/Moderate/Low based on transparency, clinical readability, and the applicability of post-hoc explanation methods such as shapley additive explanation (SHAP). We used decision curve analysis (DCA) [32] to evaluate the practical value of each model for clinical decision-making, measuring its net benefit across a range of decision thresholds. Calibration plots were used to visually demonstrate the degree of agreement between the model-predicted values and the actual observed outcomes. SHAP [33–35] was applied to unpack the black-box models, revealing feature importance levels, as shown in Figure 2. Two-sided p values less than 0.05 were considered statistically significant.

## Results

### Baseline characteristics

In the study concerning CRRT for ESKD patients, initially, 691 patients were included, and after applying strict exclusion criteria, 55 patients who did not satisfy the imposed conditions were excluded. Ultimately, 636 patients were successfully enrolled in the study, and they were subsequently divided into two major groups on the basis of the coagulation occurrence rates in their extracorporeal circulation devices: a No ECC clotting group (*n* = 437, without coagulation in their extracorporeal circulation devices) and an ECC clotting group (*n* = 199, with coagulation in their extracorporeal circulation devices).

Table 1 provides a detailed representation of the significant characteristic differences between the No ECC clotting and ECC clotting groups. Compared with the No ECC clotting group, the ECC clotting group had a greater proportion of male patients, with significant differences in heart rate, mean SysBP, ultrafiltration quantity, initial dose of LMWH, the white blood cell count, the neutrophil ratio, the lymphocyte ratio, the platelet count, hematocrit, the red blood cell count, hemoglobin, aspartate aminotransferase, alanine aminotransferase, chloride, and glucose (*p* < 0.05). However, no significant differences were found between the other variables of the two groups.

**Table 1.** Baseline characteristics of the included ESKD patients.

Characteristics	Total (*n* = 636)	No ECC clotting (*n* = 437)	ECC clotting (*n* = 199)	*p* Value
Females, n (%)	180 (28.3%)	139 (31.8%)	41 (20.6%)	0.005
Age (years)	60.89 ± 12.96	60.74 ± 12.81	61.21 ± 13.32	0.674
Weight (kg)	67.00 ± 13.71	66.37 ± 13.79	68.36 ± 13.46	0.089
Vascular access, n (%)				0.015
Dialysis arteriovenous fistulas	346 (54.4%)	227 (51.9%)	119 (59.8%)	
Internal jugular vein dialysis catheter	210 (33.0%)	160 (36.6%)	50 (25.1%)	
Femoral venous dialysis catheter	80 (12.6%)	50 (11.4%)	30 (15.1%)	
Blood dialysis filter, n (%)				0.797
ST100	417 (65.6%)	283 (64.8.%)	134 (67.3%)	
ST150	65 (10.2%)	45 (10.3%)	20 (10.1%)	
M100	154 (24.2%)	109 (24.9%)	45 (22.6%)	
Heart rate (beats/minute)	82.69 ± 17.01	81.62 ± 16.27	85.05 ± 18.33	0.018
Average systolic blood pressure (mmHg)	147.86 ± 25.92	149.33 ± 26.06	144.64 ± 25.37	0.034
Average diastolic blood pressure (mmHg)	79.31 ± 15.31	79.29 ± 14.97	79.36 ± 16.06	0.960
Treatment model (CVVH), n (%)	636 (100%)	437 (68.7%)	199 (31.3%)	0.114
Blood Flow Rate (ml/min)	182.94 ± 7.96	182.61 ± 7.81	183.67 ± 8.23	0.119
Predilution replacement fluid (ml/h)	1779.40 ± 63.00	1777.12 ± 68.59	1784.42 ± 48.29	0.175
Postdilution replacement fluid (ml/h)	537.11 ± 121.95	531.12 ± 125.95	550.25 ± 111.86	0.067
Ultrafiltration quantity (ml/h)	376.93 ± 171.97	363.68 ± 172.46	406.01 ± 167.66	0.004
Initial dose of LMWH (IU)	1579.40 ± 1060.01	1667.73 ± 1026.01	1385.43 ± 1109.25	0.002
Hourly booster dose of LMWH (IU)	62.45 ± 104.83	67.09 ± 106.56	52.26 ± 100.42	0.098
LMWH added at X h (hours)	1.62 ± 1.97	1.67 ± 1.99	1.50 ± 1.94	0.298
X-hourly booster dose of LMWH (IU)	262.11 ± 401.57	256.29 ± 374.64	274.87 ± 455.91	0.589
White blood cell (×10⁹/L)	7.71 ± 4.04	7.27 ± 3.51	8.66 ± 4.89	<0.001
Neutrophil (%)	76.74 ± 10.53	76.03 ± 10.40	78.30 ± 10.68	0.012
Lymphocyte (%)	13.76 ± 7.63	14.25 ± 7.63	12.68 ± 7.52	0.016
Red blood cell (×10^12^/L)	3.20 ± 0.77	3.16 ± 0.78	3.28 ± 0.74	0.054
Hemoglobin (g/L)	95.02 ± 22.21	93.59 ± 22.19	98.18 ± 21.99	0.016
Hematocrit (L/L)	0.30 ± 0.07	0.29 ± 0.07	0.31 ± 0.07	0.014
Platelet count (×10⁹/L)	185.71 ± 83.50	178.81 ± 79.73	200.86 ± 89.59	0.002
Platelet distribution width	16.25 ± 0.43	16.25 ± 0.43	16.25 ± 0.43	0.877
Direct bilirubin (μmol/L)	3.80 ± 11.05	3.57 ± 9.47	4.32 ± 13.92	0.428
Indirect bilirubin (μmol/L)	4.29 ± 4.30	4.17 ± 4.44	4.55 ± 3.97	0.303
Aspartate aminotransferase (U/L)	32.40 ± 81.41	25.33 ± 53.09	47.92 ± 121.23	0.001
Alanine aminotransferase (U/L)	35.00 ± 131.66	27.94 ± 85.65	50.50 ± 197.70	0.045
Alkaline phosphatase (U/L)	97.62 ± 64.39	99.70 ± 68.73	93.05 ± 53.53	0.228
Albumin (g/L)	34.56 ± 5.80	34.57 ± 5.72	34.54 ± 5.97	0.953
Blood urea nitrogen (mmol/L)	24.19 ± 10.32	24.59 ± 10.72	23.31 ± 9.35	0.147
Creatinine (μmol/L)	843.84 ± 410.52	849.78 ± 438.53	830.80 ± 341.70	0.589
Uric acid (μmol/L)	431.01 ± 166.64	435.65 ± 164.03	420.82 ± 172.24	0.298
Glucose (mmol/L)	8.34 ± 4.67	8.05 ± 4.27	8.98 ± 5.41	0.020
Calcium (mmol/L)	2.13 ± 0.27	2.11 ± 0.28	2.17 ± 0.24	0.016
Phosphate (mmol/L)	1.90 ± 0.76	1.90 ± 0.76	1.91 ± 0.77	0.841
Potassium (mmol/L)	4.70 ± 0.92	4.69 ± 0.93	4.70 ± 0.91	0.910
Sodium (mmol/L)	137.20 ± 4.30	137.29 ± 4.30	136.99 ± 4.30	0.404
Chloride (mmol/L)	100.23 ± 5.92	100.71 ± 6.05	99.18 ± 5.48	0.002
Carbon dioxide (mmol/L)	21.03 ± 5.03	20.89 ± 5.03	21.33 ± 5.01	0.304
Cholesterol (mmol/L)	3.99 ± 1.45	3.98 ± 1.38	4.03 ± 1.61	0.682
Triglycerides (mmol/L)	1.85 ± 1.45	1.84 ± 1.42	1.88 ± 1.53	0.709
High-density lipoprotein (mmol/L)	0.99 ± 0.37	0.99 ± 0.37	0.98 ± 0.38	0.870
Low-density lipoprotein (mmol/L)	2.14 ± 0.88	2.15 ± 0.86	2.14 ± 0.92	0.937
Prothrombin time (s)	13.38 ± 2.84	13.30 ± 2.46	13.57 ± 3.55	0.254
International normalized ratio	1.10 ± 0.27	1.10 ± 0.23	1.12 ± 0.35	0.300
the p-prothrombin time activity (%)	91.99 ± 21.04	92.66 ± 21.41	90.51 ± 20.18	0.233
Activated partial thromboplastin time (s)	33.20 ± 10.53	32.73 ± 8.05	34.25 ± 14.54	0.092
Fibrinogen (g/L)	6.64 ± 5.01	6.68 ± 5.13	6.53 ± 4.74	0.722
Thrombin time (s)	16.70 ± 16.57	16.17 ± 12.41	17.88 ± 23.23	0.227
D-dimer (mg/L)	3.60 ± 5.54	3.55 ± 5.72	3.69 ± 5.13	0.777

*Abbreviations***:** ECC: extracorporeal circuit clotting; LMWH: low-molecular-weight heparin; CVVH: continuous veno-venous haemofiltration.

### Data preprocessing

Missing data (3.03%) were imputed *via* multiple imputation, and SMOTE was used to balance the class distribution. Comprehensive quality control assessments revealed (1) no significant near-zero variance predictors (frequency ratio < 5 with > 10% unique values), (2) acceptable multicollinearity levels (all variance inflation factors <5), and (3) preserved distributional properties (Kolmogorov–Smirnov test: all *p* > 0.05). See Supplementary Tables S2 and S3 for details.

### Feature selection

**Figure 3.**
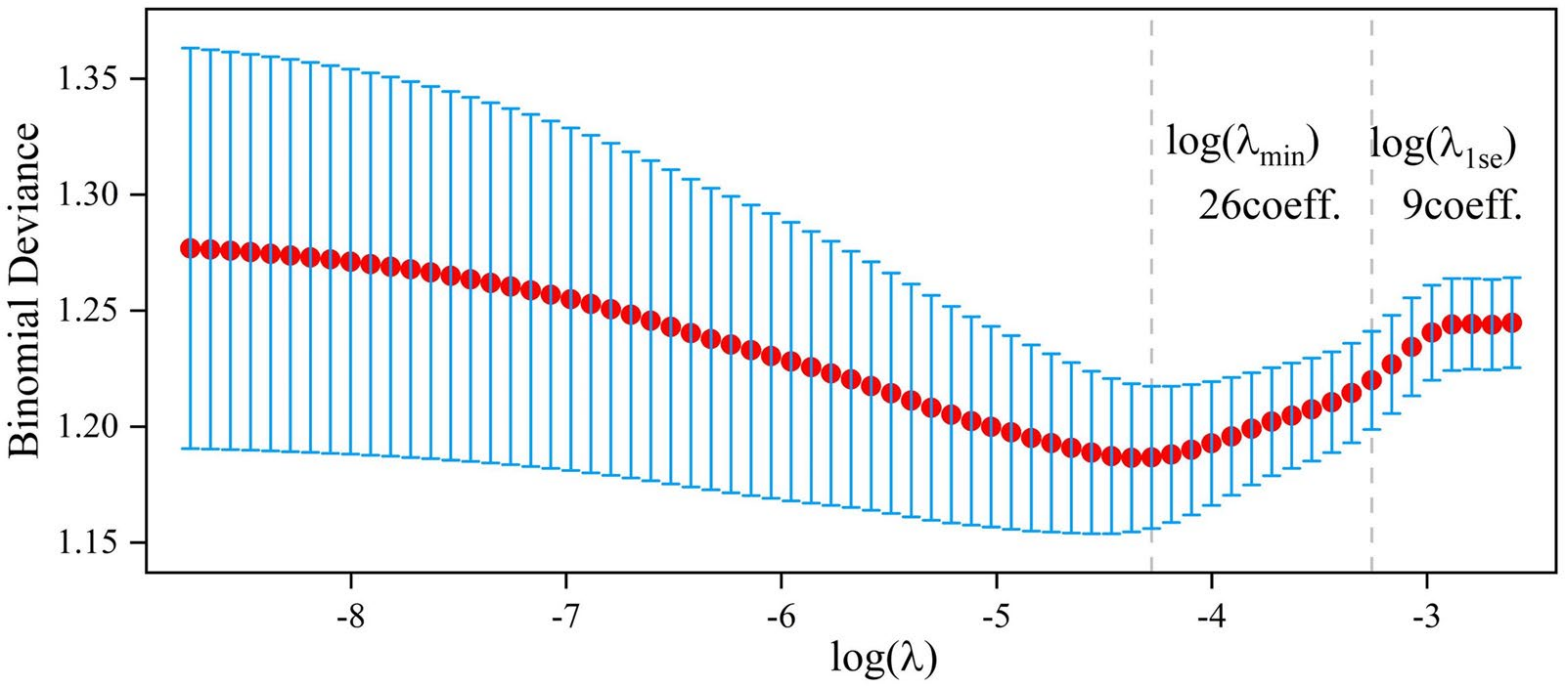
LASSO Regularization path graph. The log(λ_1se_) criterion was chosen to determine the variables used to construct models that were concise and had good predictive performance. Abbreviations: LASSO: least absolute shrinkage and selection operator.

In our study, the LASSO algorithm was employed to meticulously identify nine crucial variables (sex, ultrafiltration quantity, the initial dose of LMWH, the white blood cell count, hematocrit, the platelet count, aspartate aminotransferase, calcium, and chlorine) out of a pool of 53 candidate variables. This variable selection process aimed to enhance both the predictive performance and parsimony of our models, as shown in Figure 3.

### Model performance comparisons

**Figure 4.**
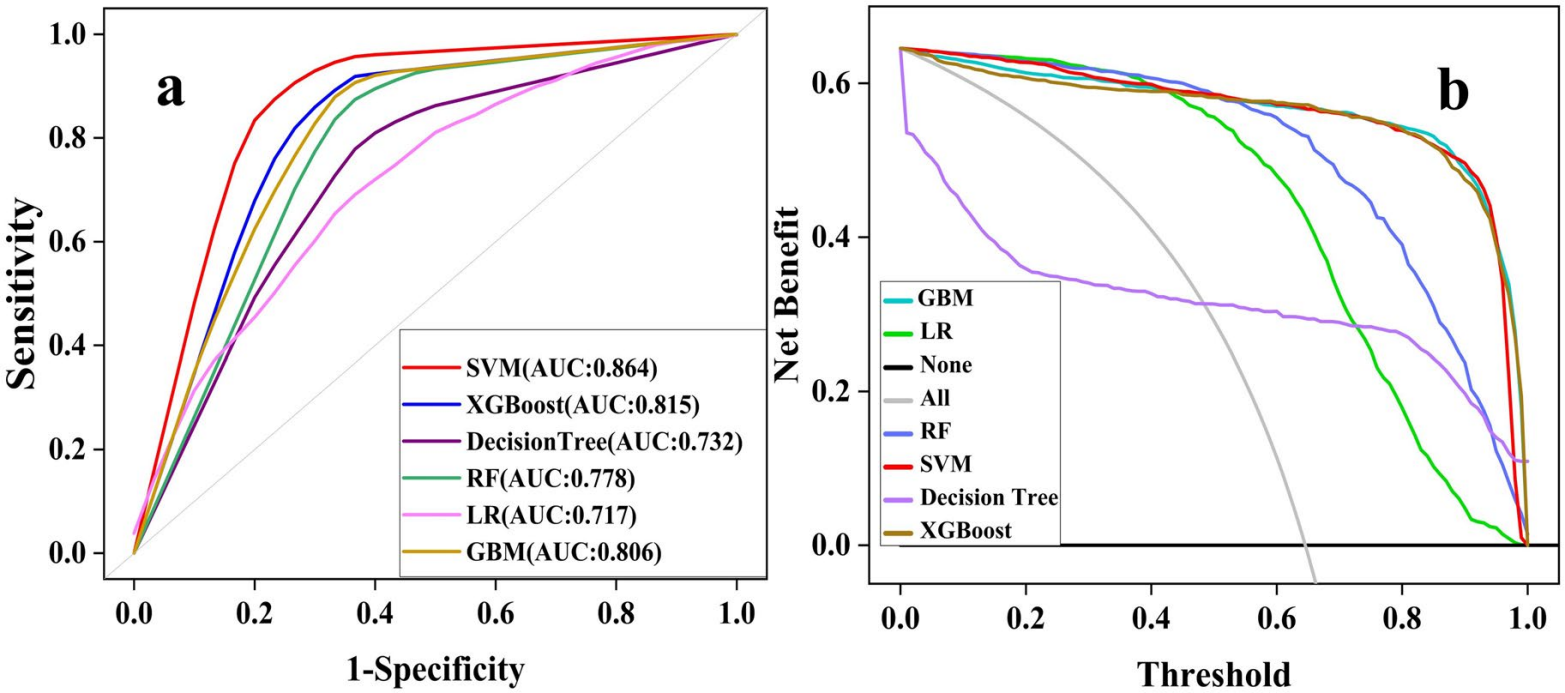
**a.** ROC curves produced by the six models. **b.** DCA results of the six models. Abbreviations: SVM: support vector machine; XGBoost: extreme gradient boosting; RF: random Forest; LR: logistic regression; GBM: gradient boosting machine; ROC: receiver operating characteristic; DCA: decision curve analysis.

We constructed six models to predict ECC during CRRT in ESKD patients and comprehensively evaluated their performance. Table 2 provides detailed descriptions of the AUC values and other performance metrics. The receiver operating characteristic (ROC) curves of all the evaluated models are shown in Figure 4a. In a comprehensive evaluation, the SVM model emerged as the top performer, with an excellent AUC value of 0.864 (0.844–0.891), coupled with remarkable scores in terms of Brier’s score of 0.076 (0.062–0.087), sensitivity of 0.780 (0.727–0.816), specificity of 0.948 (0.928–0.967), PPV of 0.897 (0.863–0.933), NPV of 0.888 (0.865–0.908), and F1 score of 0.831 (0.803–0.865). However, its intrinsic interpretability was rated as Low. It outperformed the other models overall. Following SVM, the models were ranked by AUC in descending order: XGBoost (0.815 [0.757–0.869]), GBM (0.806 [0.784–0.849]), RF (0.778 [0.753–0.799]), decision tree (0.732 [0.712–0.762]), and LR (0.717 [0.675–0.759]). Notably, while the decision tree model exhibited slightly lower sensitivity and specificity than the traditional LR model did, all five ML models significantly outperformed the LR model across key performance indicators such as the AUC, Brier’s score, PPV, NPV, and F1 score. The calibration plots of all the models, presented in Supplementary Figure 1, further reinforce the reliability and accuracy of the predictions yielded by the SVM, as this model demonstrated better calibration than the other models did. As shown in Figure 4b, the DCA revealed that the SVM model provided greater net benefits across most threshold levels.

**Table 2.** Model performance metrics.

Models	AUC (95%CI)	Brier’s score(95%CI)	Sensitivity (95%CI)	Specificity (95%CI)	PPV (95%CI)	NPV (95%CI)	F1 score (95%CI)	Interpretability
SVM	0.864 (0.844 −0.891)	0.076 (0.062 −0.087)	0.780 (0.727 −0.816)	0.948 (0.928 −0.967)	0.897 (0.863 −0.933)	0.888 (0.865 −0.908)	0.831 (0.803 −0.865)	Moderate (SHAP-enabled)
XG Boost	0.815 (0.757 −0.869)	0.118 (0.078 −0.156)	0.721 (0.630 −0.817)	0.910 (0.864 −0.950)	0.816 (0.734 −0.890)	0.857 (0.814 −0.901)	0.763 (0.685 −0.835)	Low (requires SHAP)
GBM	0.806 (0.784 −0.849)	0.162 (0.133 −0.183)	0.699 (0.655 −0.764)	0.912 (0.893 −0.928)	0.812 (0.779 −0.847)	0.849 (0.812 −0.873)	0.749 (0.722 −0.807)	Low
RF	0.778 (0.753 −0.799)	0.128 (0.123 −0.137)	0.641 (0.589 −0.675)	0.916 (0.906 −0.925)	0.806 (0.779 −0.829)	0.823 (0.805 −0.839)	0.713 (0.672 −0.741)	Moderate (feature importance)
Decision Tree	0.732 (0.712 −0.762)	0.189 (0.179 −0.200)	0.639 (0.588 −0.705)	0.825 (0.807 −0.841)	0.667 (0.649 −0.684)	0.808 (0.789 −0.839)	0.650 (0.620 −0.689)	High
LR	0.717 (0.675 −0.759)	0.198 (0.186 −0.209)	0.594 (0.563 −0.625)	0.881 (0.856 −0.906)	0.643 (0.589 −0.697)	0.723 (0.709 −0.737)	0.579 (0.543 −0.615)	High

*Abbreviations*. AUC: area under the curve; PPV: positive predictive value; NPV: negative predictive value; SVM: support vector machine; XGBoost: extreme gradient boosting; RF: random forest; GBM: gradient boosting machine; LR: logistic regression; SHAP: shapley additive explanation.

### Model explanations

**Figure 5.**
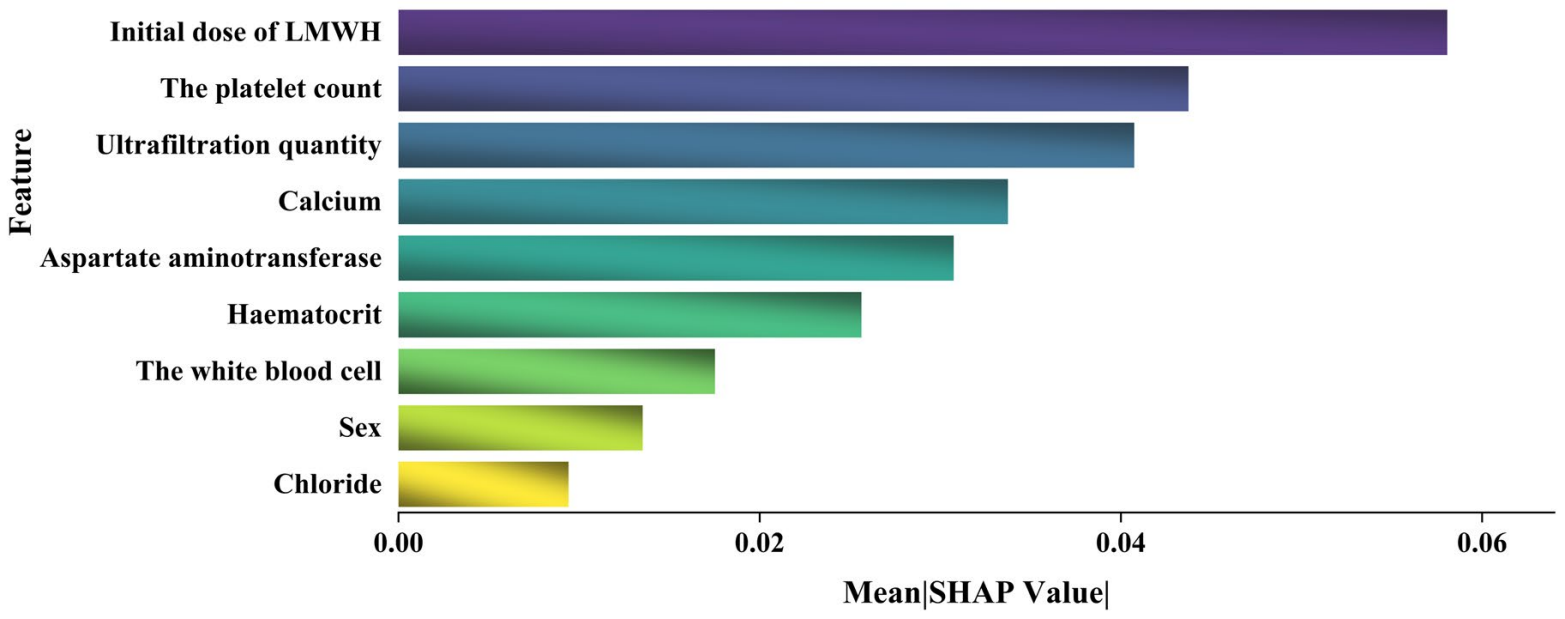
Feature importance levels derived from the SVM model. Abbreviations: LMWH: low-molecular-weight heparin.

To thoroughly investigate and quantify the importance of the features to ECC, we employed the SHAP method for feature ranking and analysis, aiming to accurately evaluate the contribution of each feature to the predictive performance of the SVM model. Figure 5, Supplementary Figures 2 and 3 shows the impact of each feature on the prediction results of the model, where the initial dose of LMWH, the platelet count, and ultrafiltration quantity are the three most significant features, with SHAP values of 0.058, 0.044, and 0.041, respectively. These findings indicate that when predicting coagulation risk during extracorporeal circulation, the initial dose of LMWH, the platelet count, and ultrafiltration quantity play crucial roles. The following features, in order of decreasing importance, are calcium, aspartate aminotransferase, hematocrit, the white blood cell, sex, and chloride.

## Discussion

Recently, several clinical prediction models for patients with ESKD and AKI have been successfully developed *via* ML technology, and these models have achieved significantly improved predictive accuracy [20,36,37]. For the first time, this study focused on patients diagnosed with ESKD and receiving CRRT, applied ML algorithms to construct multiple ECC risk prediction models, and compared their predictive performance with that of traditional LR methods. The comprehensive analysis results revealed that the ML models performed excellently in terms of discrimination, calibration, and clinical decision-making guidance. Among these approaches, the SVM model performed best, providing more predictions of coagulation risk in the extracorporeal circuit following the initiation of CRRT, with significant clinical application potential. This model can assist clinicians in identifying high-risk patients prior to initiating CRRT, thereby enabling early adjustment of anticoagulant strategies and reducing the incidence of coagulation events. For example, on the basis of the model’s predictions, doctors can individually tailor the initial dose of LMWH or ultrafiltration quantity to mitigate coagulation risk. We developed a decision-making flowchart for clinicians to visually illustrate the application of the model in individualized anticoagulation strategies during CRRT (see Supplementary Figure 4). Additionally, this model can be integrated into existing clinical workflows as part of a CRRT treatment decision support system. By inputting patients’ clinical data in real time, the model can dynamically assess coagulation risk and provide immediate decision-making recommendations to physicians, ultimately enhancing the safety and effectiveness of treatment.

During HD treatments implemented for patients with ESKD, if an urgent critical condition is observed, CRRT may be urgently needed. However, clotting in the extracorporeal circuit is a common issue encountered during CRRT. Failure to promptly identify and manage this issue may lead to treatment interruption, reduced effective treatment time, increased treatment costs, additional workload for healthcare providers, and patient blood loss with coagulation abnormalities. In severe cases, it may even result in thrombosis of the hemodialysis access [10]. Currently, the process of controlling clotting in the extracorporeal circuit during CRRT relies primarily on adjusting the dosages of anticoagulant drugs (such as LMWH, unfractionated heparin, and citrate). This is based on the patient’s weight, coagulation assessment, individual circumstances, and real-time coagulation monitoring results. However, this method poses risks of bleeding and clotting and is often based on experience, making it difficult to predict adverse clotting outcomes [5,38,39]. The formation of extracorporeal circuits during CRRT is a complex phenomenon influenced by multiple factors, and its occurrence is not limited to the dosages of anticoagulants alone. Unfortunately, there is a lack of dedicated tools for assessing coagulation risk. In light of this, developing a model capable of accurately predicting coagulation risk in a timely manner, which can be used to adjust treatment regimens on the basis of the model’s predictions, facilitating early intervention and mitigating potential losses, has become an extremely urgent and important task.

Currently, predictive models for the risk of ECC during CRRT have been gradually developed. Fu et al. [12] conducted a prospective study that included 425 patients with AKI receiving CRRT. Cox regression analysis revealed that the following key variables are associated with the occurrence of ECC: insufficient blood flow, no anticoagulation, hematocrit, lactate, and activated partial thromboplastin time. The retrospective study performed by Zhang et al. [13] focused on 170 patients who underwent CRRT without anticoagulation in intensive care units. The model they developed could identify patients whose filter lifespan was ≥24 h during non-anticoagulated CRRT. Liu et al. [40] developed a risk prediction model for ECC during CRRT *via* ML algorithms. The study included 212 patients with mixed AKI, CKD, and intensive care unit cases who did not receive anticoagulation therapy. In recent years, researchers have continued to explore various factors influencing ECC outcomes during CRRT [41]. However, the subjects in the abovementioned studies were AKI patients and patients in intensive care units, and no specific studies have been carried out on people with ESKD.

This is the first ML prediction model specifically designed to predict extracorporeal circuit clotting in ESKD patients undergoing CRRT. The differences between AKI and ESKD patients in CRRT treatment directly impact the prediction of extracorporeal circulation coagulation risks. Firstly, ESKD patients often present with chronic coagulation dysfunction, characterized primarily by platelet dysfunction and bleeding tendency. This is associated with the accumulation of uremic toxins, which impair platelet aggregation and adhesion, as well as endothelial dysfunction [42]. In contrast, AKI patients typically experience acute coagulation imbalance. Early on, they may exhibit a hypercoagulable state due to inflammatory responses, ischemia-reperfusion injury, and tissue factor exposure. Some patients, due to the rapid deterioration of renal function and accumulation of metabolic waste products, may later develop bleeding tendency [43]. Coagulation abnormalities in ESKD are more persistent and synergistically worsen the bleeding risk due to anemia, whereas coagulation changes in AKI are more dynamic and closely related to the underlying cause. Secondly, AKI patients commonly receive regional citrate anticoagulation, while ESKD patients are more likely to be managed with LMWH or no anticoagulation strategy [5,15]. Thirdly, in patients with AKI, a single dialysis session typically lasts more than 24 h, most often *via* catheter access, and is characterized by low blood flow rates and large volumes of replacement fluid, aiming to maintain hemodynamic and internal milieu stability as well as to remove inflammatory mediator [8]. In contrast, in critically ill patients with ESKD, a single dialysis session usually lasts 6–8 h, most often *via* arteriovenous fistula access, with high blood flow rates and small volumes of replacement fluid, aiming to remove uremic toxins and alleviate volume overload [24]. These differences make it challenging to directly apply AKI prediction models to ESKD patients, highlighting the clinical value of developing a dedicated prediction model for ESKD.

Among the five constructed ML models, the SVM model demonstrated the best performance. Specifically, the SVM model not only achieved the highest AUC value but also performed excellently in terms of other key performance metrics, such as Brier’s score, sensitivity, specificity, the PPV, the NPV, and the F1 score. In addition, the calibration curve exhibited a high degree of consistency between the predicted probabilities of the SVM model and the actual clotting risk of the extracorporeal circulation device, fully demonstrating the high precision and reliability of its predictive results. In the DCA, the net benefits provided by the SVM model at various risk levels were significantly better than those of the other models. This model has the ability to identify high-risk patients effectively prior to the initiation of CRRT, enabling physicians to administer targeted interventions promptly, adjust treatment protocols, and reduce the occurrence of coagulation events. The comprehensive evaluation results revealed that the SVM model performed best among the five ML models. This finding was consistent with the research results published by Zhang et al. [44] and Zhuang et al. [45] in the field of nephrology, where they built clinical prediction models for cases with small sample sizes and complex data. Moreover, the SVM model outperformed the traditional LR model in all performance metrics in this study. This is attributed to the ability of SVM to capture nonlinear interactions effectively among multiple factors through kernel functions, thereby providing strong generalizability and robustness and achieving superior performance in high-dimensional clinical data [46]. In contrast, tree-based models are prone to overfitting, whereas linear models struggle to capture complex relationships [47], and LR models have limited capability in handling such complex data [48].

SHAP was used to interpret the internal decision logic of the SVM model [35], which indicated that nine key variables are associated with the occurrence of clotting in the extracorporeal circuit of CRRT: sex, ultrafiltration quantity, the initial dose of LMWH, the white blood cells, hematocrit, the platelet count, aspartate aminotransferase, calcium, and chlorine. We collected several key machine-derived parameters, but they were not incorporated into the final model. This may be due, on the one hand, to the shrinkage property of the LASSO algorithm, which penalizes multicollinearity and low-contribution variables [29,30]. On the other hand, coagulation abnormalities specific to patients with ESKD may have weakened the predictive value of these conventional indicators [42]. The initial dose of LMWH is considered the most important indicator, which may be related to the fact that LMWH activates antithrombin and enhances anticoagulant effects. Thrombin can promote the conversion of fibrinogen into fibrin, forming thrombi; in the extracorporeal circuit, excessive thrombin activity can lead to device clotting, affecting equipment operation and patient outcomes [49]. LMWH also inhibits platelet aggregation and promotes fibrinolysis through multiple pathways, which aligns with the recommendations of the KDIGO guidelines [5], and the study conducted by Fu et al. [12] also supports this conclusion. Similarly, the platelet count was strongly associated with clotting in the extracorporeal circulation device during CRRT. This finding was consistent with the studies conducted by Zhang et al. [13] and Matthias M et al. [50], who noted that the platelet count can interact with molecules such as fibrin and vWF through processes including activation, changing shape, releasing granules, and expressing glycoprotein receptors, promoting the formation of stable thrombi, which in turn can lead to clotting in the extracorporeal circulation device. Ultrafiltration quantity has been confirmed as an important factor affecting thrombosis in extracorporeal circulation devices [51]. It can directly influence the flow state of blood and the concentration of blood components within the extracorporeal system by regulating the ultrafiltration rate, thereby impacting platelet activation, the concentrations of coagulation factors, and the interactions between blood flow and artificial surfaces, promoting or inhibiting thrombus formation. Calcium ions in serum, as key factors in the coagulation cascade process, not only activate prothrombin but also promote platelet aggregation, playing a crucial role in maintaining the stability of the coagulation mechanism. This was fully demonstrated in the research conducted by Zhang et al. [52]. Although the role of aspartate aminotransferase in the clotting of the extracorporeal circulation device has not yet been reported, this study considered it an important variable. This is mainly attributed to aspartate aminotransferase being a sensitive marker of liver injury; it is capable of indirectly reflecting the functional status of the liver, which is the primary organ for synthesizing coagulation factors and fibrinogen [53]. This finding provides clinicians with a new perspective for assessing clotting risk. Important haematological indicators, such as white blood cells and hematocrit, also play significant roles in the clotting of the extracorporeal circulation device, as has been confirmed in relevant clinical studies [12,13]. Specifically, the white blood cells significantly affect coagulation through mechanisms such as releasing procoagulant substances, influencing the platelet count functions, regulating inflammatory responses, and maintaining vascular wall integrity [54]. Hematocrit regulates coagulation conditions by adjusting the ratio of anticoagulants to whole blood and influencing calcium ion concentrations [55]. Sex differences have also been found to be important factors affecting clotting risk. Research published by Coleman et al. [56] revealed that women have a greater risk of thrombosis than men do. Although chlorine ions do not directly participate in the biochemical reactions of coagulation, they indirectly affect the coagulation mechanism by regulating the osmotic pressure and acid–base balance of the extracellular fluid, thereby impacting the clotting performance of the extracorporeal circulation device [57].

This study made a certain contribution by constructing a model for predicting ECC risk in patients with ESKD during CRRT, using an individualized DCA to optimize the clinical decision-making process, and providing scientific support for treatments. However, this research has several limitations. First, as this study is a single-center design and did not utilize local citrate anticoagulation strategies, this limits the external validation and generalizability of the model. Additionally, blood transfusion, medication use, and underlying conditions during the CRRT process may influence the results. However, due to the retrospective design and incomplete documentation of clinical variables, these factors were not included in the analysis. Future research should include prospective, multicenter external validation and optimization, incorporate additional clinical variables, and thereby further enhance the translational impact of the study and increase its citation potential. Second, model performance is constrained by data quality and variable complexity. Although the multiple imputation method is used for clinical variable processing, there is still a risk of bias. Future efforts are needed to optimize preprocessing procedures to mitigate the impact of noise. Third, the model has not yet been applied in clinical practice. To further promote the practical application of the constructed SVM model, subsequent research could focus on developing a web-based calculator or a mini-program. This tool can assist clinicians in rapidly assessing coagulation risk by inputting relevant variables, thereby enabling targeted interventions on key influencing factors. Fourth, the focus of this study was solely on coagulation risk, and we did not analyze clinical outcomes such as mortality. Future endeavors should broaden its scope to encompass other complications and risk factors associated with CRRT. Additionally, dynamic data should be incorporated to enable real-time predictions. Furthermore, it is essential to stay at the forefront of development in the field by running the ECC risk model in parallel with AKI risk prediction models to achieve a more comprehensive risk assessment. Additionally, integrating advanced algorithms, such as personalized anticoagulant dosing algorithms, to dynamically optimize anticoagulation strategies will contribute to a more complete risk assessment framework that fully supports clinical decision-making.

## Conclusion

This study revealed that the SVM model exhibited remarkable performance in terms of predicting the risk of extracorporeal circuit coagulation in ESKD patients after initiating CRRT, significantly outperforming the LR and other ML models. Concurrently, our research revealed that the initial dose of LMWH and the platelet count are pivotal factors influencing ECC. The DCA and SHAP methods provide strong support for clinical decision-making and herald the broad application prospects of ML for predicting ESKD-related complications and optimizing treatments.

## Supplementary Material

Supplemental Material

Supplemental Material

Supplemental Material

Supplemental Material

## Data Availability

The datasets used and/or analyzed during the current study are available from the corresponding author upon reasonable request.
