# Supplementary figures and images for: Machine learning model predicts clotting risk during CRRT in ESKD patients: a SHAP-interpretable approach
Source: Ren Fail. 2025 Oct 9;47(1):2562448. doi: 10.1080/0886022X.2025.2562448 (PMC12517423; doi:10.1080/0886022X.2025.2562448)

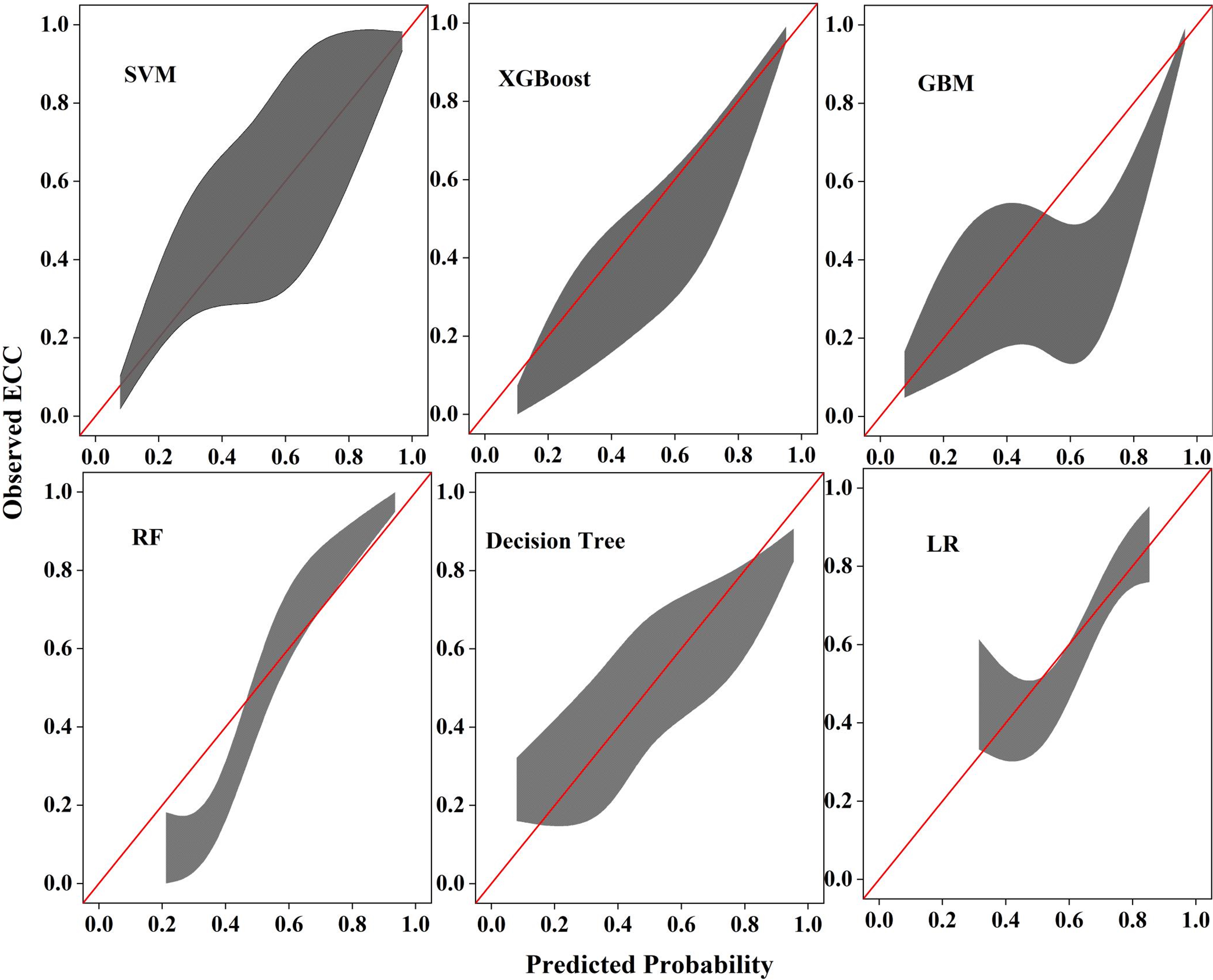

Supplement: Supplemental Material [file IRNF_A_2562448_SM5122.jpg]
